# The anti-inflammatory effects of three different dietary supplement interventions

**DOI:** 10.1186/s12967-025-07167-x

**Published:** 2025-10-16

**Authors:** Amrita Vijay, Liz Simpson, Melanie Tooley, Sarah Turley, Afroditi Kouraki, Anthony Kelly, Cristina Menni, Josh Armstrong, Shann Jones, Ana M Valdes

**Affiliations:** 1 NIHR Nottingham Biomedical Research Centre and Academic Unit of Injury, Recovery and Inflammation Science, School of Medicine, University of Nottingham, Nottingham, UK; 2 School of Life Sciences, University of Nottingham, Nottingham, UK; 3 School of Agriculture and Food Sciences, University College Dublin, Dublin, Ireland; 4 Department of Twin Research and Genetic Epidemiology, King’s College London, London, UK; 5 Department of Pathophysiology and Transplantation, Università Degli Studi di Milano, Via Francesco Sforza, 35, 20122 Milan, Italy; 6 Angelo Bianchi Bonomi Hemophilia and Thrombosis Center, Fondazione IRCCS Cà Granda Ospedale Maggiore Policlinico, 20122 Milan, Italy; 7 Chuckling Goat Ltd, Wales, UK

## Abstract

**Background:**

Understanding how diet influences inflammation requires identifying specific dietary components responsible for anti-inflammatory effects. This study examined the impact of six-week supplementation with a single-source prebiotic fibre (inulin), omega-3, or a synbiotic (fermented kefir + prebiotic fibre mix) on a broad range of inflammatory markers.

**Methods:**

Serum inflammatory proteins were profiled using the Olink 96 inflammation panel in a 6-week intervention. Participants received one of the following: synbiotic (*n* = 20; 170 ml kefir + 10 g prebiotic), omega 3 (*n* = 33; 500 mg/day), inulin fibre (*n* = 31; 20 g/day), or no supplementation (*n* = 20 control). Changes from baseline and between groups were analysed using parametric methods and effect sizes (Cohen’s d). FDR-adjusted *p* < 0.05 was considered significant.

**Results:**

All three dietary interventions significantly reduced inflammatory markers versus control. TNF-α decreased with omega-3 (d= − 0.618, 95% CI -0.73 to -0.09, *p* = 0.01) and inulin fibre (d=–1.012, 95% CI -0.71 to -0.20, *p* = 0.001). The synbiotic group showed broader and larger reductions, including IL-6 (d=–0.882,95% CI -1.36 to -0.17, *p* = 0.01), IFN-γ (d=–0.940, 95% CI -2.03 to -0.31, *p* = 0.009), SIRT2 (d=–1.505, 95% CI -1.30 to -0.51, *p* < 0.0001), 4EBP1 (d=–1.384, 95% CI -1.43 to -0.32, *p* = 0.0004), CCL23 (d=–1.356, 95% CI -1.40 to -0.48, *p* = 0.0002), and mucosal cytokines CCL25 (d=–1.137, 95% CI -0.90 to -0.23, *p* = 0.001) and CCL28 (d=–1.006, 95% CI -0.80 to -0.16, *p* = 0.003). Increases in serum butyrate correlated with reductions in IL-6 following the synbiotic intervention.

**Conclusions:**

All interventions reduced systemic inflammation, but the synbiotic produced broader and stronger effects, targeting proteins linked to immune and metabolic function. While gut microbiome profiling was not included in this study, it is planned in future work to clarify how synbiotics may influence host–microbiome interactions and inflammatory regulation.

**Trial registration:**

Trial registration Clinicaltrials.gov NCT06480812. Registered 28th June 2024 Retrospectively registered https//clinicaltrials.gov/study/NCT06480812.

**Graphical abstract:**

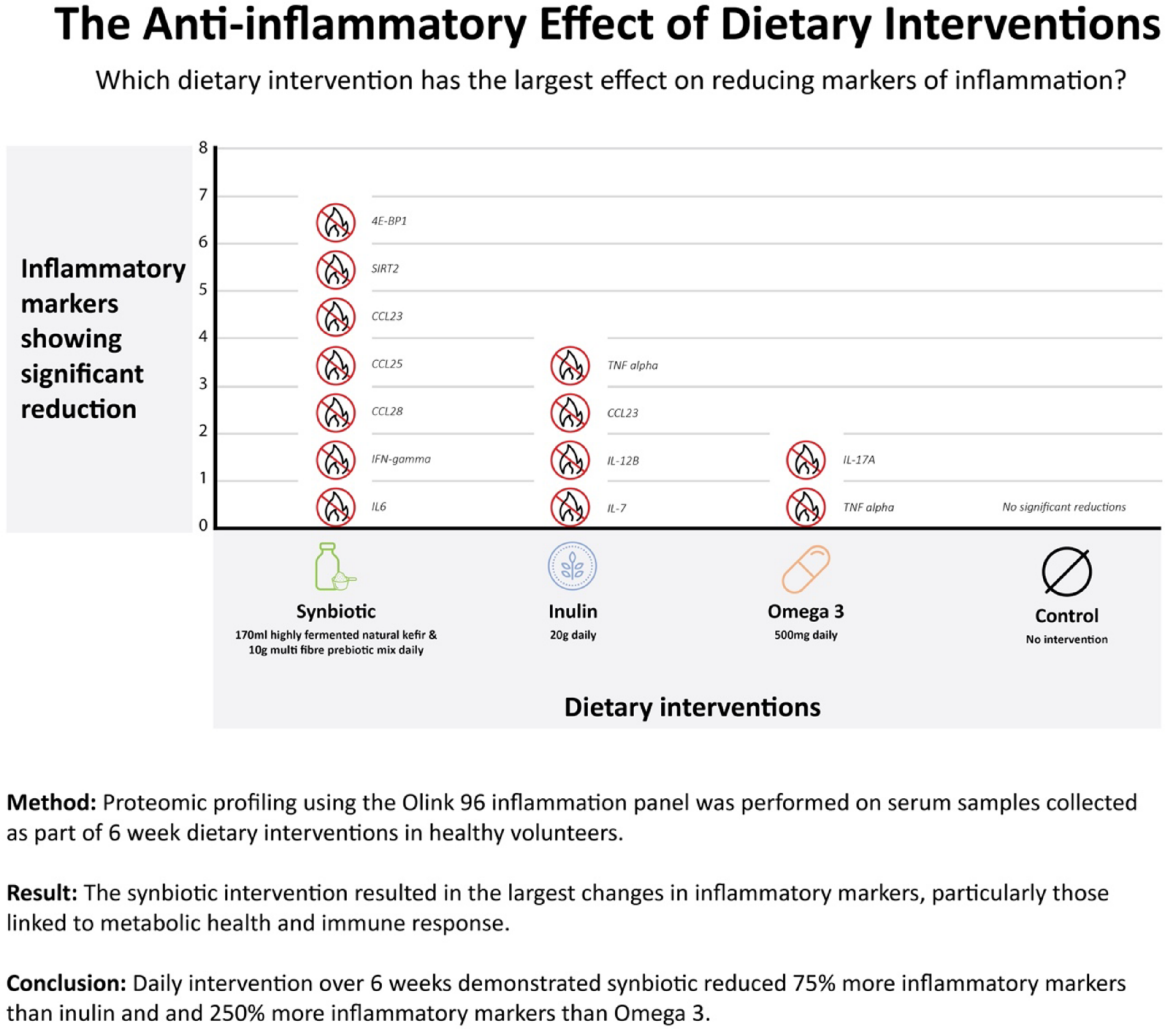

**Supplementary Information:**

The online version contains supplementary material available at 10.1186/s12967-025-07167-x.

## Introduction

Nutritional components like prebiotics, omega-3 fatty acids, and synbiotics have the potential to modulate the immune system and reduce chronic inflammation. Understanding how diet can modulate inflammation involves dissecting dietary components to ascertain which specific elements contribute to any anti-inflammatory effects. For example, prebiotics are non-digestible fibres that selectively stimulate the growth and/or activity of beneficial gut bacteria, which can influence immune function and inflammation [1, 2], and Omega-3 fatty acids, particularly EPA (eicosapentaenoic acid) and DHA (docosahexaenoic acid), are known for their anti-inflammatory properties [3, 4].

Synbiotics refer to a combination of live microorganisms and substrates that are selectively utilised by the host’s gut microbiota to provide a health benefit [5]. The prebiotic component is specifically designed to support the probiotic organism, promoting its survival and adherence within the gastrointestinal tract. The principle underpinning this concept is that the simultaneous utilisation of probiotics and prebiotics promotes a synergistic interaction, maximising health benefits for the host beyond what either could achieve independently [6, 7].

Fermented foods such as kefir naturally contain live probiotics from the fermentation process offering a diverse range of health benefits [8, 9]. Specifically, the microbial community of kefir consists of a diverse and complex blend of mainly lactic acid bacteria (LAB), alongside acetic acid bacteria and various yeasts [10] which together produce essential metabolites, including peptides, exopolysaccharides, amino acids, vitamins and short chain fatty acids [11]. These compounds not only contribute to kefir’s distinctive flavour and aroma but also underpin its numerous health benefits.

Regular consumption of the above products have been shown to have anti-inflammatory effects [6, 12] and in the case of fermented foods, to modulate immune responses [13]. By assessing biomarkers like C-reactive protein (CRP), interleukin-6 (IL-6), and tumour necrosis factor-alpha (TNF-α), alongside clinical outcomes, researchers can gauge the efficacy of these supplements in reducing inflammation, which could lead to improved health outcomes and quality of life for individuals suffering from inflammatory diseases.

The current study aimed to compare the anti-inflammatory effects of three different dietary interventions: an isolated prebiotic fibre (Inulin), anti-inflammatory Omega 3 and a synbiotic (combination of a fermented drink and a prebiotic fibre mix) by evaluating their impact on biomarkers of inflammation and associated health outcomes.

## Methods

### Study population and interventions

**Fig. 1:**
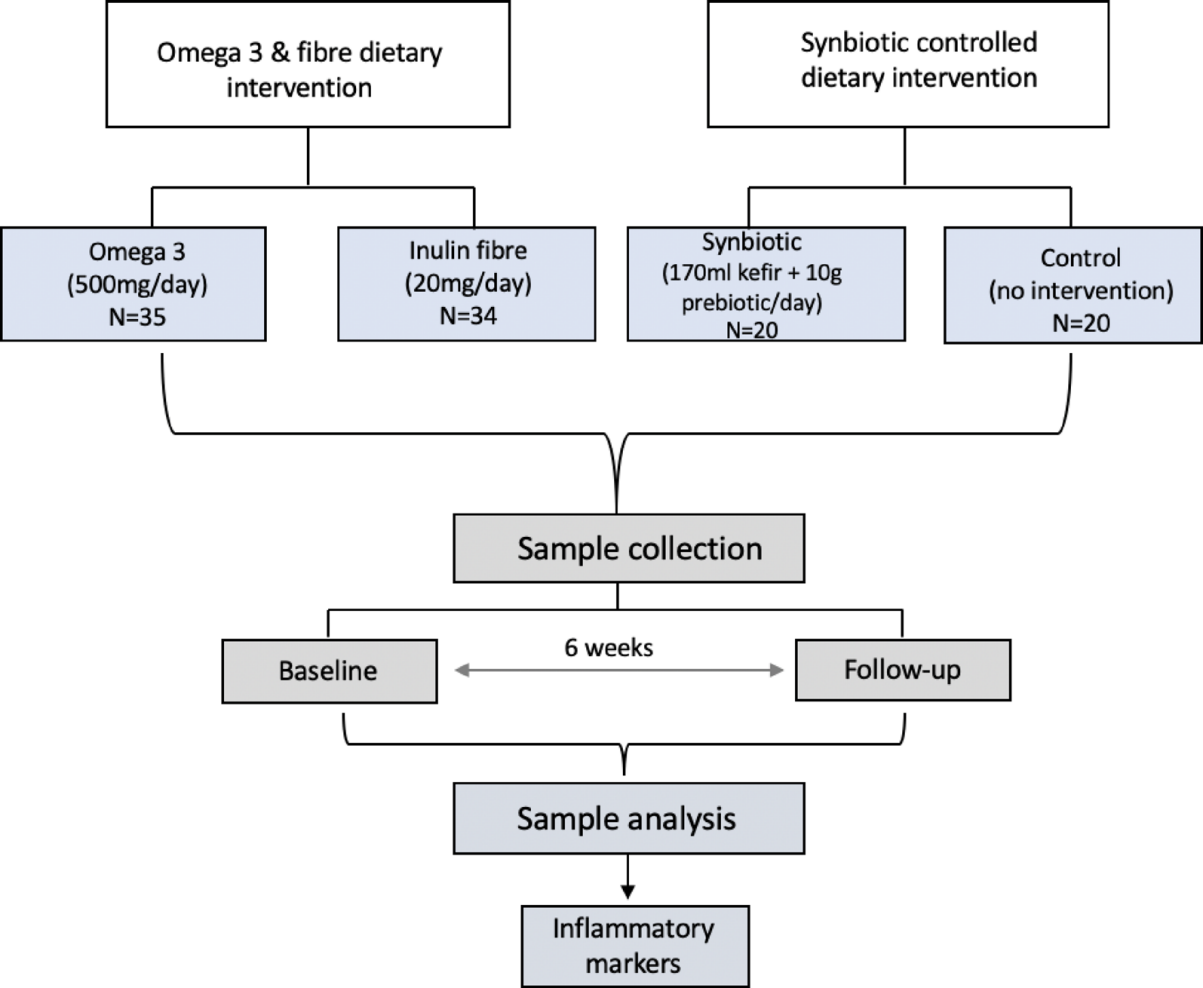
Schematic flow chart of dietary interventions and study design

**Fig. 2:**
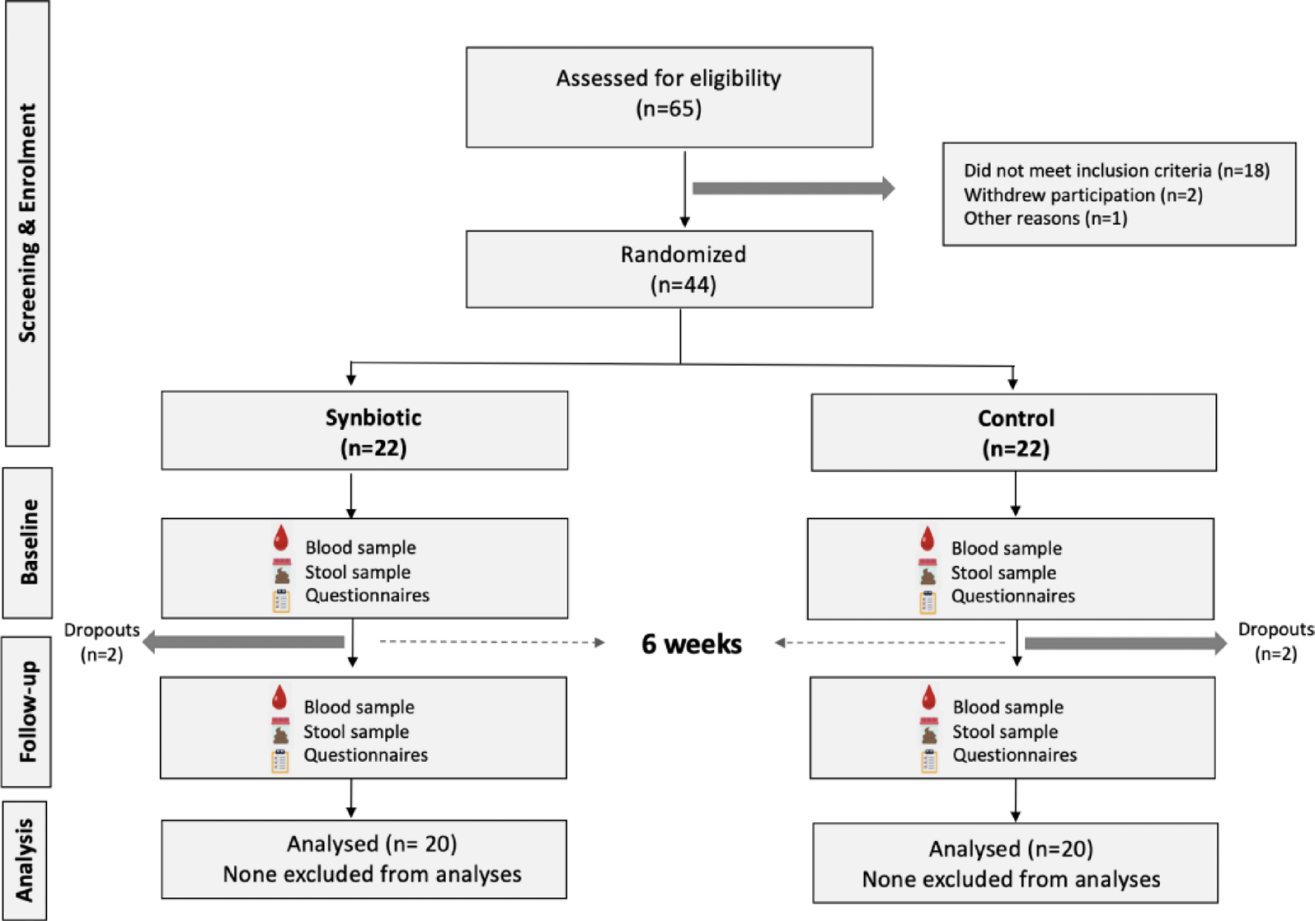
Consort diagram summarising study recruitment and design for the synbiotic intervention study

We analysed study participants from 3 different dietary interventions along with a control group of participants (Fig. 1). This included (i) 64 participants from the Omega 3 and inulin fibre intervention study who were recruited from the TwinsUK registry, as previously described [4] (ii) 40 participants from a randomised controlled trial designed to explore the effect of synbiotic supplementation on systemic inflammation and metabolic health (Fig. 2). Post hoc power analyses were conducted using G*Power software (version 3.1.9.6) to estimate the achieved statistical power based on observed effect sizes, sample sizes, and a two-tailed alpha level of 0.002.

**Omega 3 and fibre intervention study**: Participants were randomised either into the Omega 3 arm (500 mg of Omega 3 supplements (165 mg EPA + 110 mg DHA) or inulin fibre arm (20 g inulin fibre) daily for 6 weeks and were equally allocated between treatment arms (allocation ratio 1:1) with at least *n* >32 in each arm. Participant eligibility included those aged >18 y who had a body mass index (BMI) between 20 and 39.9 kg/m^2^ and had a low habitual fibre consumption (< 15 g/d). Participant screening, inclusion and exclusion criteria have been described in detail previously [4].

**Synbiotic randomised controlled intervention**: This study was conducted at the University of Nottingham, and participants were recruited using the Nottingham post and social media platforms. 40 participants in total were randomised into either the synbiotic or control arms (Fig. 2). The synbiotic arm were given 170 ml kefir and 10 g of a prebiotic mix (supplied by Chucking Goat Ltd), consumed as a smoothie daily for 6 weeks. Individuals in the control arm were provided with no intervention to follow for 6 weeks but were given the synbiotic at the end of the trial period. The goat’s milk kefir contains 27 naturally occurring live bacterial cultures [14] and the prebiotic mix consists of 18 different types of prebiotics including arabinoxylan and cellulose/hemicellulose from psyllium husk, beta glucans, chitin and mannan from maitake mushroom, arabinan from quinoa, fructooligosaccharides (FOS) from beetroot, glycyrrhizins and glycyrrhizin from liquorice root, isomaltooligosaccharide (IMO) from miso, pectin from orange peel, xylan and galactan from spirulina, resistant starch from arrowroot, inulin from chicory, xyloglucan from tamarind, xylo oligosaccharides (XOS) from rice bran, guar bean, and galactooligosaccharides (GOS) from chickpeas. Participant eligibility included those aged >18 y who had a body mass index (BMI) between 20 and 39.9 kg/m2. Individuals were excluded if they met any of the following criteria: had any gastrointestinal condition or food intolerance (e.g. IBS, malabsorptive conditions such as IBD, coeliac), lactose intolerance, diabetes mellitus or any condition with an inflammatory component such as asthma, psoriasis, arthritis etc., were taking fibre or probiotic foods/ supplements, were taking any prescribed medication, were following or anticipated to commence a specialised commercially available weight loss diet and/or program, were pregnant or breastfeeding, or had a diagnosed psychiatric illness.

Neither participants nor researchers were blinded to the above interventions. The participants were booked in for baseline and a follow-up visit at the end of the 6-week intervention period. Randomisation was performed using an online software (www.sealedenvelope.co.uk). All participants provided written informed consent.

### Ethics

The Omega 3 and inulin fibre trial was approved by the West Midlands Black Country Research Ethics Committee (18/WM/0066) and is registered under the clinicaltrials.gov database (NCT03442348) and the Synbiotic controlled intervention trial was approved by the University of Nottingham Faculty of Medicine & Health Sciences Research Ethics Committee approval (FMHS 314–0623) and registered under the clinicaltrials.gov database (NCT06480812).

### Sample collection

Blood, stool samples and anthropometric measures were collected at both baseline and follow-up visits as part of the omega 3 and inulin fibre trial as well as the synbiotic controlled intervention study. Fasting blood samples were collected from participants using standard procedures. Details of the sample collection and storage procedure for the Omega 3 and inulin fibre trial have been described previously [4]. For the synbiotic-controlled intervention, venous blood was collected from participants into 5 ml Serum Separation Tubes (Vacutainer, BD). Samples were left to stand at room temperature for 15 min before being centrifuged for 10 min at 3000 RCF. The resulting serum was aliquoted into 2 ml cryo-vials and stored immediately at -80 C. Anthropometric measures (height, weight and BMI) were collected at baseline and follow-up visits as part of all intervention trials.

### Sample analysis

#### Inflammatory markers

Inflammatory markers from fasting serum samples across all intervention groups were performed by Olink Bioscience (Uppsala, Sweden & Leeds, UK) using the Olink Target 96 panel, a multiplex immunoassay panel using PEA technology (https://olink.com/products/olink-target-96*).*

#### Cardiometabolic markers

Lipid markers (Cholesterol, HDL, LDL, non-cholesterol HDL, Triglycerides), HsCRP, insulin and glucose were measured from fasting serum samples using the Siemens Adviva 1800 High-Density ELISA kits by Affinity Biomarkers Ltd (London, UK). Homeostatic Model Assessment of Insulin Resistance (HOMA-IR) index was calculated from fasting insulin and glucose concentrations using the following formula: HOMA-IR = Insulin (mIU/L) x Glucose (mmol/L) / 22.5.

#### Short-chain fatty acids

Targeted short-chain fatty acid (SCFA) profiling was performed on the serum samples from the synbiotic study at the Edinburgh Mass Spec Core Facility, University of Edinburgh using liquid chromatography coupled with high-resolution mass spectrometry (LC-HRMS) as described previously [15]. Following derivatisation with 3-nitrophenylhydrazine, the SCFAs measured were; acetate, propionate, butyrate, iso-obutyrate, valerate and iso-valerate.

### Statistical analysis

#### Association between nutritional interventions and changes in inflammatory proteins

Fold changes in serum inflammatory protein levels from baseline to follow-up were calculated for each dietary intervention and the control groups. These changes were visualised using volcano plots, with the log2 fold change (LFC) on the x-axis and the corresponding statistical significance (-log10 p-value) on the y-axis.

To compare changes from baseline to follow-up, paired *t*-tests were used for all measures that did not violate assumptions of normality. Unpaired t-tests were used to measure significant differences between intervention and control groups. To achieve normality, most biomarkers were log10-transformed. For measures whose distributions were not normal, non-parametric methods, specifically Wilcoxon matched-pairs signed rank tests, were carried out. One-way ANOVA was used to assess differences in continuous demographic variables between each intervention group and the control, while the Chi-Square test was used to analyse categorical demographic variables. For inflammatory markers, the probability of a reduction from baseline was modelled using logistic regression (binomial logit), with intervention group as the exposure and age included as a covariate. To compare the effect of dietary supplementation vs. control, we computed the change on any given measure from baseline to follow-up (Δ) for each individual for each measure. When comparing the dietary interventions to the control arm, the effect size was quantified using Cohen’s delta (*d*) with the corresponding 95% CI. Spearman correlations were performed to identify the association between changes in short-chain fatty acids and inflammatory markers. FDR adjusted *p* < 0.05 was considered statistically significant. Analyses were performed using GraphPad Prism 10.2.0.

## Results

The descriptive characteristics of the two interventional cohorts, including the control group, are shown in Table S1. The synbiotic study, consisting of both the synbiotic and control groups, recruited participants aged 18 years and older. In contrast, the omega-3 and inulin fibre intervention study enrolled older individuals, specifically those aged 60 years and above. We carried out statistical comparisons to assess whether differences existed in baseline demographic characteristics between each intervention group and the control group. The results showed no statistically significant differences in age, sex, or body mass index (BMI) between the synbiotic group and the control group (Table S1). However, participants in the omega 3 and inulin fibre intervention groups were older than those in the control group. To address the potential confounding influence of age on changes in inflammatory markers, subsequent analyses were all adjusted for age as a covariate.

**Fig. 3:**
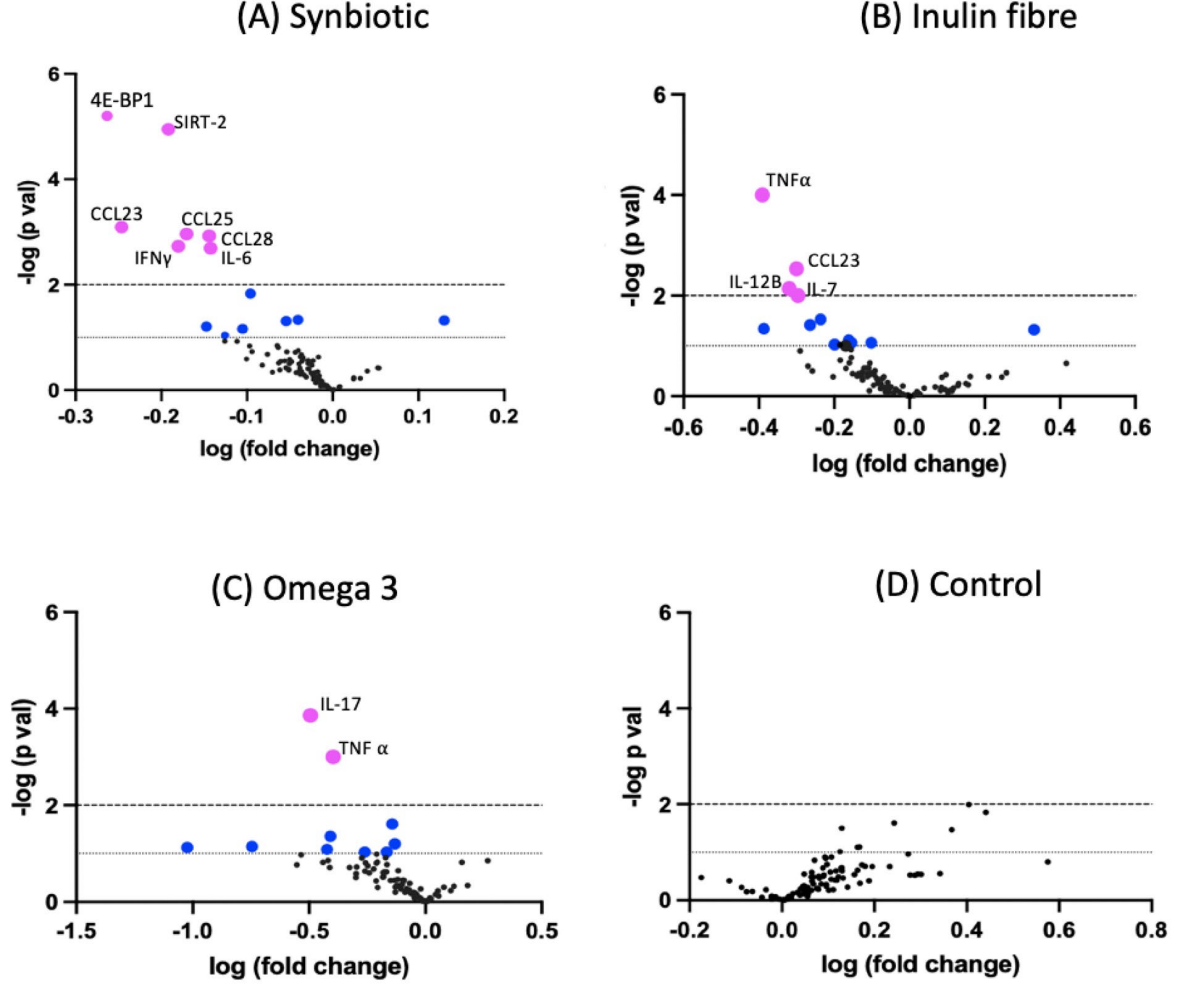
Volcano plots of significant inflammatory markers across dietary intervention groups (**A**–**C**) and the control (**D**). Each plot shows the relationship between statistical significance (-log p-value) and log fold change in inflammatory markers. Pink dots represent proteins with -log p-values > 2, surpassing the FDR threshold. Blue dots indicate significant proteins that do not meet the FDR correction, while black dots are non-significant. The dotted line represents the FDR-adjusted significance threshold (*p* < 0.003, -log p-value = 2)

Serum levels of 92 inflammatory proteins were quantified using the Olink platform across all dietary interventions, including the control group. The synbiotic intervention elicited a broader and more pronounced reduction in inflammatory markers compared to the other interventions, including both systemic inflammatory proteins and mucosal chemokines. These findings indicate anti-inflammatory effects at both systemic and tissue-specific levels (Fig. 3).

Specifically, seven inflammatory proteins were found to be altered in the synbiotic intervention (all FDR p val < 0.003). These included pro-inflammatory cytokines such as IL-6 and INF gamma but also other specific proteins that are known to play key roles in inflammation, immune response, and metabolic regulation. For example, 4EBP1 (Eukaryotic translation initiation factor 4E-binding protein 1) is a key regulator of protein synthesis that can influence cellular growth and inflammation, while SIRT2 (Sirtuin 2), a therapeutic target in viral infections but is also involved in controlling oxidative stress and promoting metabolic balance. The chemokines CCL23 (C-C motif chemokine ligand 23), CCL25 (C-C motif chemokine ligand 25), and CCL28 (C-C motif chemokine ligand 28) are linked to immune cell recruitment and gut mucosal inflammation, with their reductions suggesting improved immune regulation and reduced systemic inflammation. These changes however, were not observed in the inulin fibre or omega-3 intervention groups, highlighting the unique immunomodulatory potential of the synbiotic intervention.

We carried out unpaired t-tests between the select significant inflammatory proteins from each intervention and the control group and found the largest effect sizes corresponding to inflammatory proteins in the synbiotic arm (SIRT2 *d*= -1.505; p = < 0.0001), 4EBP1 *d*= -1.384; *p* = 0.0004, CCL23 *d*=-1.356; *p* = 0.0002), as shown in.

**Fig. 4:**
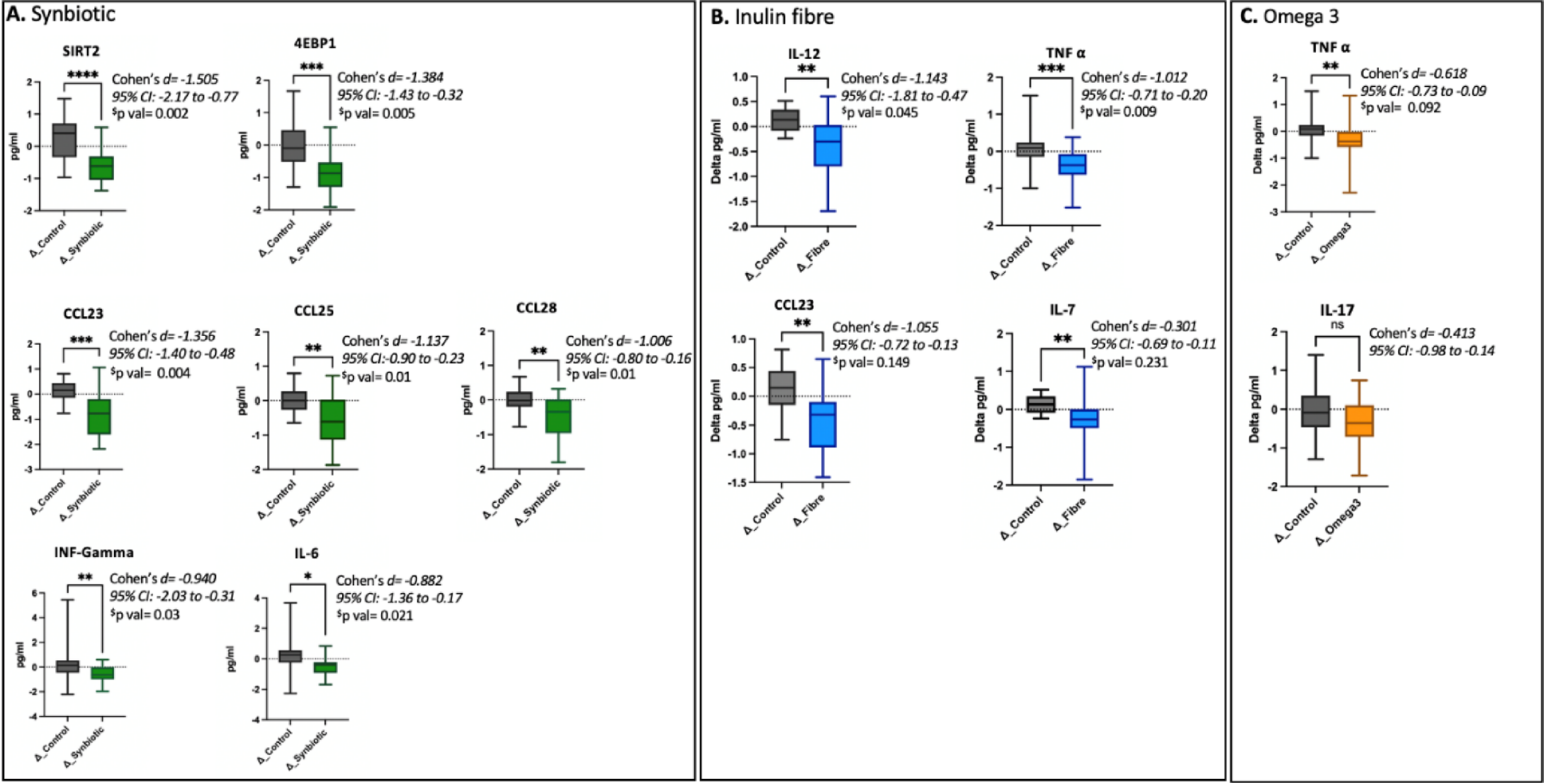
Box-and-whisker plots showing changes in inflammatory marker concentrations following the three dietary supplement interventions. Panels represent unadjusted changes in circulating inflammatory markers between control and intervention groups for: **A** Synbiotic, **B** Inulin fibre, and **C** Omega-3 supplementation. Each box-and-whisker plot displays the median (horizontal line), interquartile range (box), and minimum to maximum values. Changes are plotted in delta values representing pre-to-post intervention differences. Significant differences between groups (unadjusted) are indicated by asterisks (*p < 0.05, **p < 0.01,***p < 0.001,****p < 0.0001) and *Cohen’s d* effect sizes quantify the magnitude of change. 95% confidence intervals are reported for effect size estimates. “ns” denotes non-significance. ^$^p values are adjusted for age as a covariate

Given that the synbiotic dietary intervention led to changes in a broader range of inflammatory proteins compared to inulin fibre or omega-3 supplementation alone, we investigated whether the inflammatory proteins with the largest effect sizes were associated with markers of metabolic health. To do this, we compared changes in lipid profiles, glucose, and insulin levels in response to the synbiotic intervention versus the control. The 6-week synbiotic intervention resulted in significant reductions in total cholesterol, LDL, and non-HDL cholesterol (Table S2). However, no significant correlations were observed between changes in inflammatory proteins and changes in lipid markers. In addition, we did not see any significant differences in glucose, insulin or HOMA IR (Table S2).

**Fig. 5:**
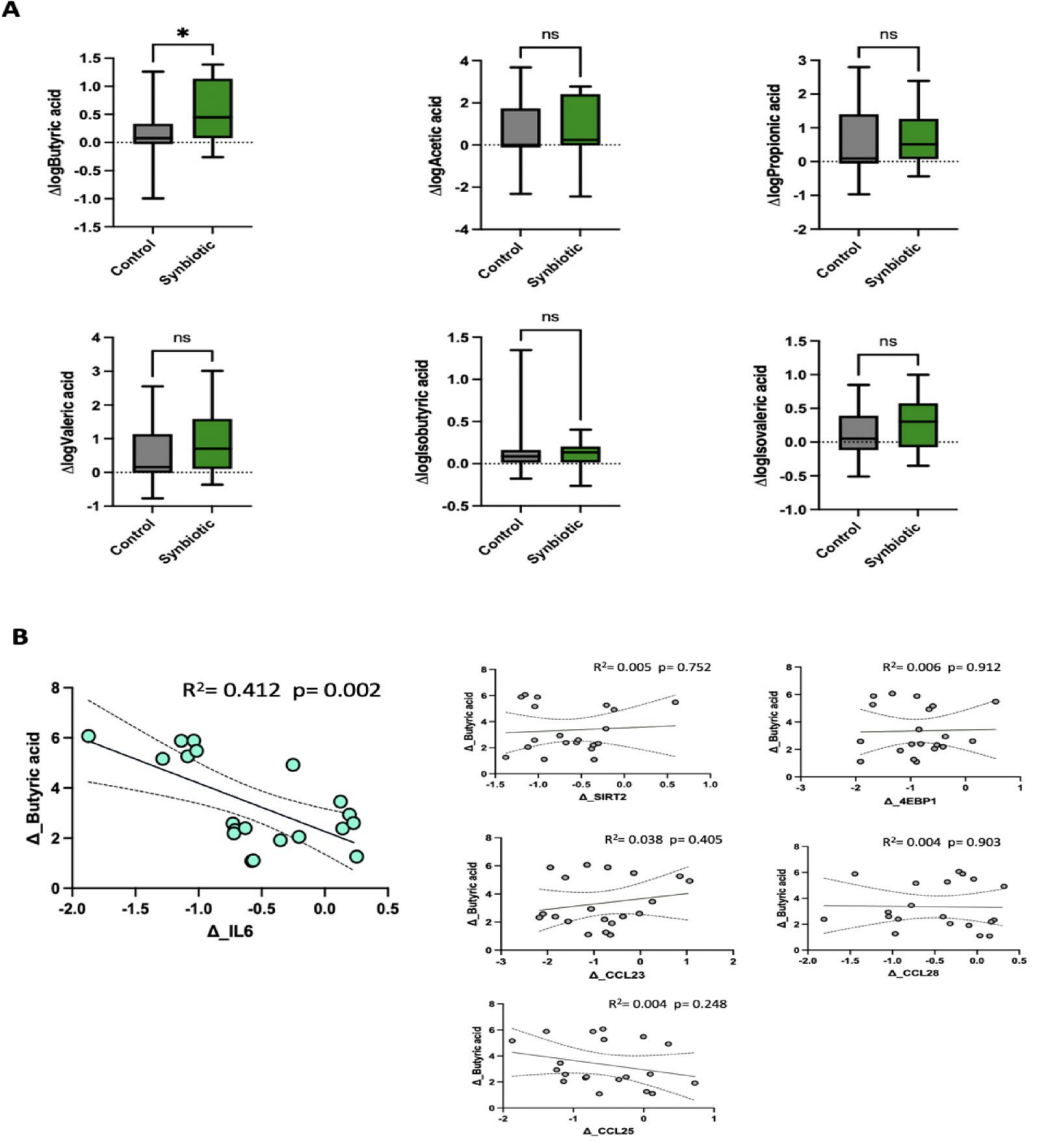
Impact of synbiotic supplementation on short-chain fatty acids (SCFAs) and their relationship with inflammatory markers. **A** Box-and-whisker plots showing changes in log-transformed serum SCFA concentrations (Δ log SCFA) following synbiotic intervention compared to control. **B** Scatter plots show correlations between changes in butyric acid and changes in inflammatory markers (Δ IL-6, Δ SIRT2, Δ 4EBP1, Δ CCL23, Δ CCL25, and Δ CCL28). Solid lines represent linear regression fits, and dashed lines indicate 95% confidence intervals. Statistical significance is indicated by asterisks (*p < 0.05), while “ns” denotes non-significance

We then decided to investigate the effects of the synbiotic intervention in the context of SCFAS and hypothesized that changes in SCFAs might correlate with alterations in inflammatory markers. Over the 6-week period, the synbiotic intervention led to increases in all measured SCFAs (Supplementary Fig. 2). Among these, butyric acid was increased in the synbiotic group compared to the control (*p* = 0.01, 95% CI 0.07 to 0.58) (Fig. 5A), suggesting a specific response to the intervention. Furthermore, the increase in butyric acid was correlated with a decrease in the pro-inflammatory marker IL-6 (*r* = -0.57, *p* = 0.01, Fig. 5B), reinforcing the role of butyrate in modulating systemic inflammation. However, no correlations were observed between butyrate and any of the other inflammatory markers, suggesting alternative mechanistic pathways which are yet to be explored.

## Discussion

In the current study, we investigated the effects of various dietary interventions, specifically inulin fibre, omega-3 fatty acids and synbiotics, on systemic inflammatory profiles, using serum inflammatory proteins as biomarkers. The synbiotic intervention resulted in reductions across a broader range of inflammatory proteins and demonstrated larger effect sizes compared to inulin fibre or omega-3 supplementation alone. Notably, decreases were observed in seven inflammatory proteins (4EBP1, SIRT2, CCL23, CCL25, CCL28, IL-6, and INF-gamma). These findings highlight the potential of synbiotic supplementation to modulate inflammation and improve metabolic health, contributing to the growing body of evidence supporting the role of fermented foods containing probiotics in regulating the gut-immune axis [8, 13, 16].

The key inflammatory proteins exhibiting the largest changes provide insight into the inflammatory pathways modulated by the synbiotic intervention. For example, SIRT2, a member of the sirtuin family, plays a pivotal role in regulating metabolism, inflammation, and ageing [17–19]. Beyond its well-established role in metabolic and inflammatory disorders, such as hepatic fibrosis [20] and vascular complications [21], SIRT2 has also been implicated in anti-viral responses [22]. Recent studies have demonstrated that SIRT2 is involved in modulating the replication and transcription of various viruses, including hepatitis B virus (HBV), through interactions with specific transcription factors. Furthermore, pharmacological inhibition of SIRT2, has shown promise in reducing viral replication and impairing viral transcriptional activity, suggesting its potential as a therapeutic target in viral infections [23]. 4EBP1, a key effector of the mTOR signalling pathway, regulates cellular growth and energy balance [24, 25]. The significant association between its reduction and BMI decrease observed in response to the synbiotic intervention suggests that 4EBP1 may be a crucial link between inflammation and metabolic health and may serve as a biomarker for inflammation-associated metabolic dysfunction. Its role in regulating energy expenditure and nutrient availability highlights the importance of targeting this pathway for therapeutic benefits in metabolic disorders. The decrease in CCL23, a chemokine involved in immune modulation and tumour progression [26] were observed in response to both the synbiotic as well as the fibre supplementation. However, the effect size was much greater in the synbiotic intervention further highlighting its strong anti-inflammatory potential.

In addition, we also observed slightly weaker changes with regard to controls in the following mucosal chemokines CCL25 and CCL28. These are both mucosal chemokines that are commonly present in the lining of the digestive tract and are primarily associated with gut lining inflammation [27]. CCL25 has been shown to play an important role in a variety of inflammation-related diseases, such as cardiovascular disease (CVD), rheumatoid arthritis, hepatitis, inflammatory bowel disease, and asthma. Both CCL25 and CCL28 activity is upregulated in IBD/ active colitis and correlates very strongly with inflammation of the gut lining [28, 29]. Finally, we saw significant decreases in markers of systemic inflammation in response to inulin fibre and omega 3, which we have reported previously [30].

While inulin fibre and omega 3 supplementations also showed anti-inflammatory effects, their impact was less pronounced than that observed with the synbiotic supplementation for the same 6-week period. Our findings are consistent with previous studies, such as those by Wastyk et al. [13], which showed that consumption of fermented foods- rich in probiotics and synbiotics elicited more robust changes in immune phenotypes and inflammatory markers compared to single source prebiotics fibres alone.

The current findings highlight the unique benefits of kefir, a naturally fermented food that contains an array of live microbes and beneficial microbial metabolites (postbiotics) that collectively contribute towards immune modulation [31]. Unlike isolated supplements, kefir provides a combination of beneficial live microbes, short-chain fatty acids, and bioactive peptides [10]. This in combination with a diverse prebiotic fibre mix, synergistically enhances its anti-inflammatory effects, as we have reported in the current study. These effects may also be mediated through interactions between the gut microbiome and the host immune system. Prebiotic fibres selectively promote the growth of SCFA-producing microbes, such as butyrate producers [32, 33], while kefir introduces microbial diversity that may enrich functional capacity [31, 34]. Butyrate, in particular, is known to support gut barrier integrity, promote regulatory T cell differentiation, and suppress pro-inflammatory cytokines including IL-6 [35] as well as play a key role in mitigating intestinal inflammation [36]. These gut–immune interactions may help explain the broader range of inflammatory markers modulated by the synbiotic intervention compared to omega-3 and inulin fibre alone. The current synbiotic combination may therefore serve as a naturally accessible, non-pharmacological and cost-effective dietary intervention to reduce a broader range of inflammatory markers, including both systemic and specific/localised inflammatory markers.

The current study has several limitations. First, each intervention has a relatively small sample size, which, although comparable to other similar studies [13] may limit the generalisability of the findings. Additionally, while post hoc power analyses indicated that most inflammatory markers were adequately powered these analyses are retrospective and do not substitute for prospective power calculations performed during study design. We acknowledge the absence of a true placebo control in the current study design. While this may limit our ability to fully isolate the specific effects of the intervention from placebo, the primary outcomes were objective blood-based biomarkers, which are less prone to placebo effects than subjective measures. We also acknowledge that the absence of participant and investigator blinding may have introduced bias in the assessment and reporting of outcomes. Future studies employing a double-blind design would strengthen the methodological rigour and reduce the potential for such bias.

While we observed systemic anti-inflammatory effects, we did not analyse the direct alterations in the gut microbiome composition in response to the dietary interventions. As the gut microbiome plays a critical role in modulating the immune system, we wish to include metagenomic analysis to better understand how these interventions specifically alter microbial communities and their relationship with the observed changes in inflammatory markers as the next steps. In addition to exploring taxonomic differences, we will also investigate functional changes such as shifts in carbohydrate-active enzymes (CAZymes), short-chain fatty acid synthesis pathways, and other microbial metabolic functions which may offer deeper insight into the mechanisms underlying nutritional modulation of host immunity.

Thirdly, exploring changes in immune phenotypes, gene expression profiling and metabolomics would provide valuable insights into the specific actions of probiotics and prebiotics on immune function. Finally, whilst the control and synbiotic (kefir plus prebiotic fibre mix) intervention were well matched for all demographic characteristics, the omega-3 and inulin fibre interventions were carried out in an older demographic. For this reason, we had to adjust results for age but it is possible that this might have reduced our power to detect additional inflammatory markers that decrease compared to controls. This is based on the fact that many markers of systemic inflammation increase with age, hence our analysis might be underestimating effects by comparing drops in older people with a nutritional intervention to lack of change in younger people without. Moreover, age-related immune changes particularly immunosenescence and altered inflammatory regulation may influence the magnitude and variability of response to dietary interventions, potentially masking or modulating the effects observed. It is also important to note that our study was not powered to conduct subgroup analyses by age, and any such analyses would be underpowered and exploratory at best. Despite these limitations, we still observed significant changes across all three interventions.

The current study adds to the growing body of evidence supporting the use of fermented foods and synbiotics in modulating the gut-immune axis. It highlights the potential of designing targeted dietary interventions to influence systemic inflammation and metabolic health, opening avenues for future research into the specific microbiome-mediated mechanisms that underpin these effects.

## Supplementary Information

Supplementary Material 1

## Data Availability

The datasets during and/or analysed during the current study available from the corresponding author on reasonable request.
