# Supplementary material for: The anti-inflammatory effects of three different dietary supplement interventions
Source: J Transl Med. 2025 Oct 16;23:1081. doi: 10.1186/s12967-025-07167-x (PMC12529832; doi:10.1186/s12967-025-07167-x)
Supplement: Supplementary file 1 — Supplementary Material 1 [file 12967_2025_7167_MOESM1_ESM.docx]

# Supplementary tables and figures

**Table S1**: Descriptive characteristics of intervention and control groups


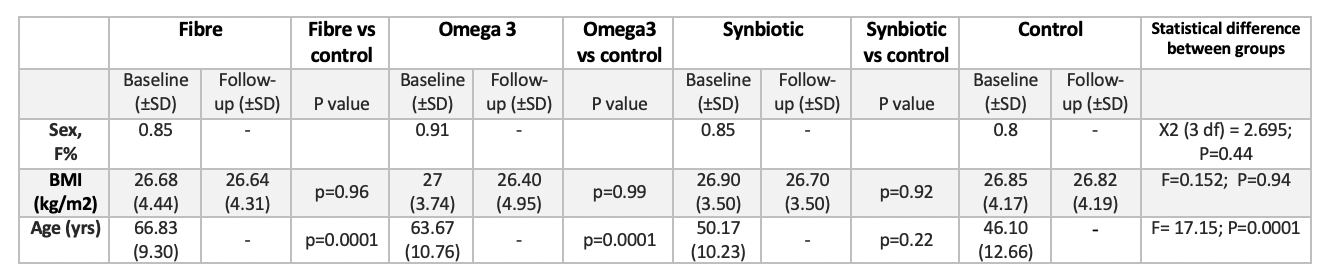


**Table S2:** Cardiometabolic markers in the intervention (synbiotic) and control arms

|  | Synbiotic (n=20) | | | Control (n=20) | | |
| --- | --- | --- | --- | --- | --- | --- |
|  | Baseline | Follow-up |  | Baseline | Follow-up |  |
|  | Mean  (Std dev) | Mean  (Std dev) | P value | Mean  (Std dev) | Mean  (Std dev) | P value |
| Total cholesterol (mmol/L) | 5.74 (1.31) | 5.38 (1.25) | **0.040** | 4.75 (1.01) | 4.72 (0.81) | 0.964 |
| Triglycerides (mmol/L) | 1.17 (0.91) | 1.06 (0.85) | 0.168 | 1.04 (0.42) | 0.99 (0.43) | 0.978 |
| HDL (mmol/L) | 1.87 (0.43) | 1.81 (0.46) | 0.654 | 1.71 (0.39) | 1.75 (0.39) | 0.188 |
| LDL (mmol/L) | 3.98 (1.35) | 3.62 (1.28) | **0.018** | 3.06 (0.95) | 2.98 (0.75) | 0.577 |
| Non-HDL (mmol/L) | 3.87 (1.35) | 3.58 (1.25) | **0.014** | 3.04 (0.92) | 2.94 (0.65) | 0.791 |
| Glucose (mmol/L) | 5.36 (0.48) | 5.37 (0.39) | 0.953 | 5.12 (0.52) | 5.11 (0.39) | 0.992 |
| Insulin (mIU/L) | 8.65 (5.69) | 8.20 (5.25) | 0.242 | 8.29 (3.76) | 8.88 (4.59) | 0.277 |
| HOMA IR | 2.14 (1.66) | 2.01 (1.48) | 0.409 | 1.909 (0.90) | 1.973 (1.02) | 0.430 |

**Table S3:** Glucose, insulin and HOMA IR levels in the Omega 3 and Inulin fibre intervention arms

|  | Omega 3 | | | Fibre | | |
| --- | --- | --- | --- | --- | --- | --- |
|  | Baseline (SD) | Follow up (SD) | P value | Baseline (SD) | Follow-up (SD) | P value |
| Glucose (mmol/l) | 4.07 (0.58) | 4.04 (0.62) | 0.982 | 4.29 (0.64) | 3.97 (0.37) | 0.263 |
| Insulin (mIU/L) | 6.86 (3.88) | 7.03 (3.61) | 0.575 | 7.31 (3.23) | 6.53 (3.37) | **0.009** |
| HOMA IR | 1.22 (0.65) | 1.24 (0.70) | 0.608 | 1.43 (0.62) | 1.18 (0.62) | 0.077 |

**Table S4:** Post hoc power analysis for the intervention groups

| Intervention group | Variable | Effect size with 95%CI  (Cohen’s *d*) | Post hoc power | FDR adjusted p value (α) |
| --- | --- | --- | --- | --- |
| Synbiotic | SIRT2 | -1.50 (-2.17 to -0.77) | 95.4% | <0.003 |
|  | IL6 | -0.88 (-1.36 to -0.17) | 91.9% | <0.003 |
| Omega 3 | TNFα | -0.61 (-0.73 to -0.09) | 91.5% | <0.003 |
|  | IL-17 | -0.41 (-0.98 to -0.14) | 83.0% | <0.003 |
| Fibre | IL-12 | -1.14 (-1.81 to -0.47) | 92.9% | <0.003 |
|  | IL-7 | -0.30 (-0.69 to -0.11) | 50.9% | <0.003 |

**Figure S1:**


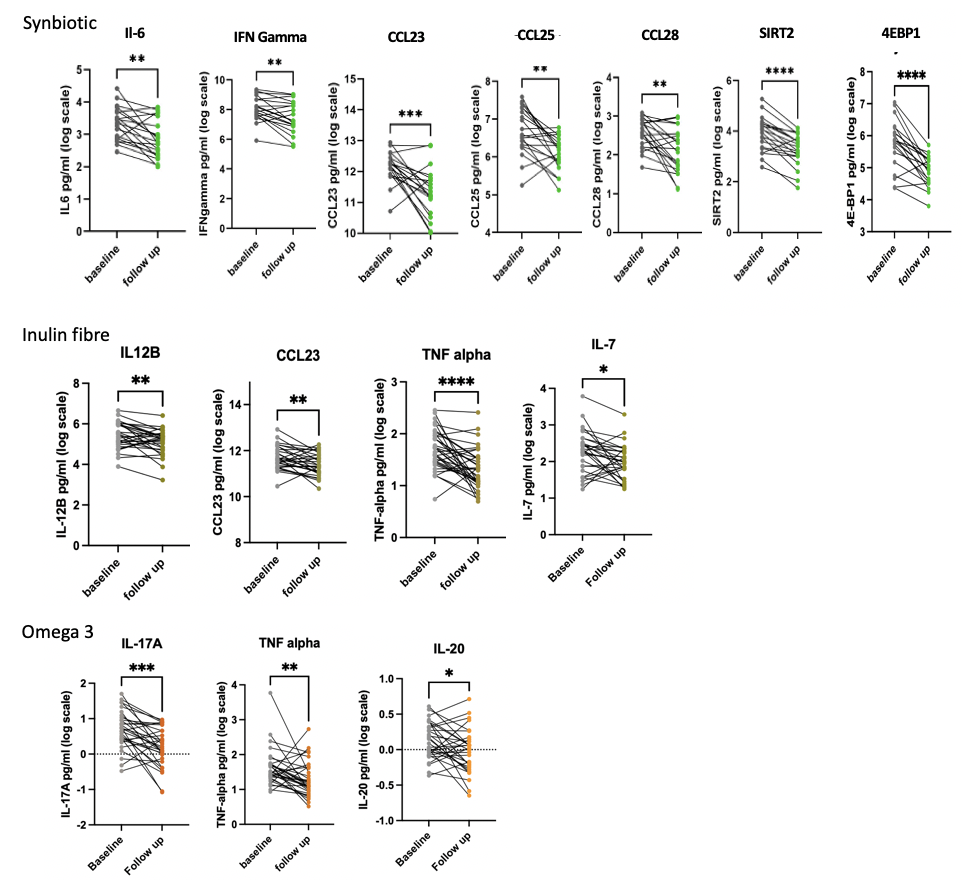


Change in significant inflammatory markers between baseline and follow-up in the different dietary supplementation interventions. Statistically significant differences are indicated by asterisks: (*p < 0.05, **p < 0.01,***p < 0.001,****p < 0.0001).

**Figure S2:**

**
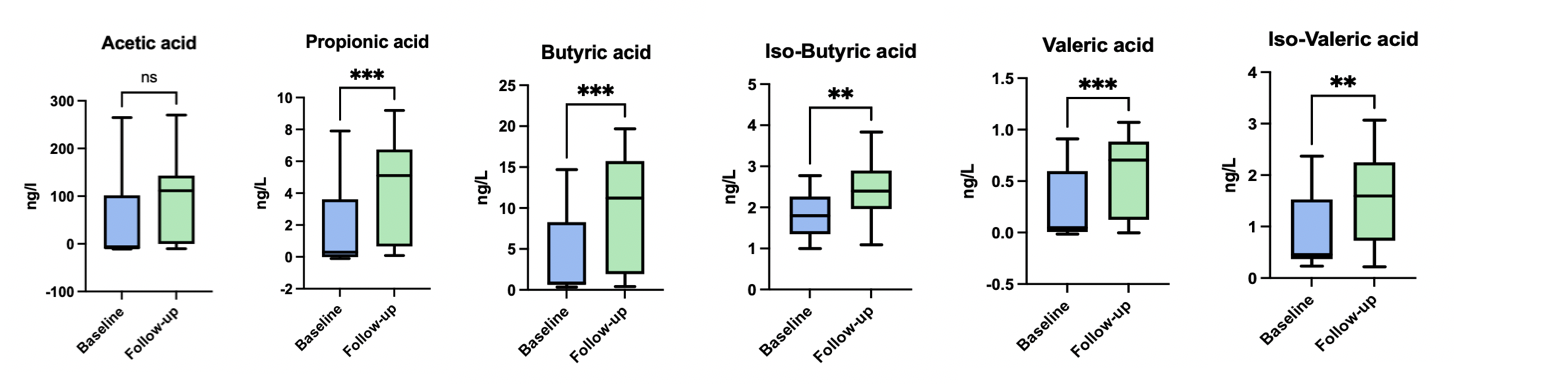
**

Change in SCFA concentrations between baseline and follow-up in the intervention group. Statistically significant differences are indicated by asterisks: (*p < 0.05, **p < 0.01,***p < 0.001,****p < 0.0001).

Figure S3:


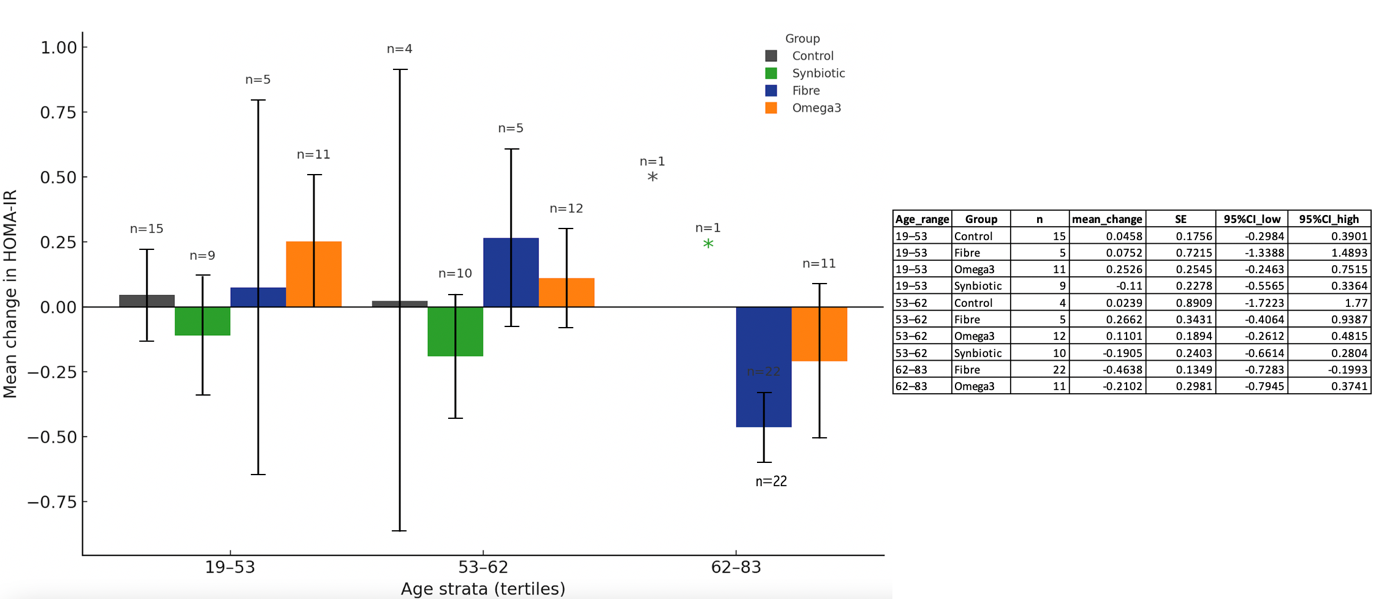


Mean change in HOMA-IR by age tertile and intervention. Bars show the group mean change from baseline to follow-up; error bars denote the standard error of the mean (SE) and are shown only when n ≥ 2. The value above each bar is the sample size; categories with n = 1 are marked with an asterisk. Summary table listing n, mean change, SE and 95% CI used to derive the error bars.
